# bFGF-Loaded PDA Microparticles Enhance Vascularization of Engineered Skin with a Concomitant Increase in Leukocyte Recruitment

**DOI:** 10.3390/bioengineering13010110

**Published:** 2026-01-16

**Authors:** Britani N. Blackstone, Zachary W. Everett, Syed B. Alvi, Autumn C. Campbell, Emilio Alvalle, Olivia Borowski, Jennifer M. Hahn, Divya Sridharan, Dorothy M. Supp, Mahmood Khan, Heather M. Powell

**Affiliations:** 1 Department of Materials Science and Engineering, The Ohio State University, Columbus, OH 43210, USA; blackstone.38@osu.edu (B.N.B.); everett.199@buckeyemail.osu.edu (Z.W.E.); campbell.2285@buckeyemail.osu.edu (A.C.C.); 2 Division of Basic and Translational Research, Department of Emergency Medicine, The Ohio State University, Columbus, OH 43210, USA; syed.baseeruddinalvi@osumc.edu (S.B.A.); divya.sridharan@osumc.edu (D.S.); mahmood.khan@osumc.edu (M.K.); 3 Davis Heart and Lung Research Institute, The Ohio State University, Columbus, OH 43210, USA; 4 Department of Biomedical Engineering, The Ohio State University, Columbus, OH 43210, USA; alvalle.1@buckeyemail.osu.edu (E.A.); borowski.26@buckeyemail.osu.edu (O.B.); 5 Shriners Children’s Ohio, Dayton, OH 45404, USA; hahnjf@ucmail.uc.edu (J.M.H.); suppdm@ucmail.uc.edu (D.M.S.); 6 Department of Surgery, University of Cincinnati College of Medicine, Cincinnati, OH 45267, USA; 7 Center for Stem Cell & Organoid Medicine (CuSTOM), Cincinnati Children’s Hospital Medical Center, Cincinnati, OH 45229, USA

**Keywords:** polydopamine, basic fibroblast growth factor, drug release, dermal template, engineered skin, vascularization

## Abstract

Engineered skin (ES) can serve as an advanced therapy for treatment of large full-thickness wounds, but delayed vascularization can cause ischemia, necrosis, and graft failure. To accelerate ES vascularization, this study assessed incorporation of polydopamine (PDA) microparticles loaded with different concentrations of basic fibroblast growth factor (bFGF) into collagen scaffolds, which were subsequently seeded with human fibroblasts to create dermal templates (DTs), and then keratinocytes to create ES. DTs and ES were evaluated in vitro and following grafting to full-thickness wounds in immunodeficient mice. In vitro, metabolic activity of DTs was enhanced with PDA+bFGF, though this increase was not observed following seeding with keratinocytes to generate ES. After grafting, ES with bFGF-loaded PDA microparticles displayed dose-dependent increases in CD31-positive vessel formation vs. PDA-only controls (*p* < 0.001 at day 7; *p* < 0.05 at day 14). Interestingly, ES containing PDA+bFGF microparticles exhibited an almost 3-fold increase in water loss through the skin and a less-organized basal keratinocyte layer at day 14 post-grafting vs. controls. This was associated with significantly increased inflammatory cell infiltrate vs. controls at day 7 in vivo (*p* < 0.001). The results demonstrate that PDA microparticles are a viable method for delivery of growth factors in ES. However, further investigation of bFGF concentrations, and/or investigation of alternative growth factors, will be required to promote vascularization while reducing inflammation and maintaining epidermal health.

## 1. Introduction

Large total body surface area, full-thickness wounds pose a unique challenge for treatment as viable donor sites for skin grafts are limited and repeat harvesting results in scarring [1,2]. Through isolation and expansion of autologous cells, tissue-engineered skin (ES) can significantly decrease the amount of donor skin needed, covering as much as 100 times the size of the initial donor area [3], and heal the wound with minimal scarring [4]. As most models of engineered skin do not contain a vascular plexus, as human autograft skin does, neo-vascularization must occur rapidly to prevent ischemia, necrosis, and infection [5,6,7]. Lack of a vascular plexus delays vascularization of ES, resulting in reduced engraftment rates compared with autograft [4]. Clinically, challenges associated with the early lack of vasculature were abrogated by irrigating cultured skin substitutes with a topical solution of antimicrobials and nutrients three times a day for the first 5–7 days post-grafting [3,8]. While effective, this process is extremely labor-intensive.

Efforts to improve the rate of vascularization in engineered skin fall into two general categories: formation of vascular networks in vitro [9,10,11,12,13,14,15,16,17,18,19,20] or delivery of pro-angiogenic factors, such as vascular endothelial growth factor (VEGF) [21], platelet-derived growth factor (PDGF), and basic fibroblast growth factor (bFGF), to speed neovascularization in vivo [22]. Cultured endothelial cells (EC) within 3D bioprinted hydrogels [10,12,13,16,17,18] and sponges [15] spontaneously form vascular networks via self-assembly within two weeks [10,12,13,15,16,17,18]. Anastomosis of these in vitro-generated vascular networks with host vascular networks generally occurred by seven days post-grafting [10,11,14], with perfusion observed within three weeks [10,11,12,13]. Pre-vascularization via co-culture of ECs with the dermal component of the engineered skin has been studied [15,16,19,20]; however, the addition of EC to the culture was shown to decrease epidermal organization and barrier function [16,20] and required 4 weeks for perfusion [16], which is a similar timeframe to the standard engineered skin model without ECs [7,23,24].

While pre-vascularized engineered skin constructs display therapeutic potential, their clinical effectiveness is currently overshadowed by lengthy in vitro culture, including the requirement for primary culture of autologous dermal microvascular endothelial cells. Pro-angiogenic growth factors, bFGF, VEGF, and angiopoietin-1, significantly improved the rate of tubule formation in vitro [22,25,26] and angiogenesis in vivo [22,27,28] when delivery was localized to the wound area via microspheres and scaffolds. As a potent fibroblast mitogen and stimulator of keratinocyte migration, bFGF has been shown to increase proliferation of human endothelial cells [29] and upregulate VEGF mRNA expression in vascular smooth muscle cells [30]. Further, bFGF has been used to improve wound closure and epithelialization in patients suffering from burn wounds [31], diabetic foot ulcers [32,33], and pressure ulcers [34,35]. When bFGF was topically applied to wounds, repeated high-dosage applications were necessary to elicit favorable clinical outcomes [31,33,34,35,36]. However, bFGF is rapidly cleared from tissue with some studies reporting a half-life of less than two hours [37,38], assessed by serum levels in human patients.

To provide temporal and spatial control of release, these growth factors can be loaded into hydrogels [39,40,41,42], scaffolds [43,44,45,46], and micro-/nanoparticles [22,46,47,48]. As polydopamine (PDA) can easily adsorb growth factors and other pharmaceuticals and are intrinsically adhesive to tissue due to their catechol, amine, and quinone structures [49], they are frequently used for drug delivery. PDA is non-cytotoxic [50,51,52], degradable [52], an excellent reactive oxygen species scavenger [50,52,53,54,55], and can be used to coat scaffolds and wound dressings [56,57,58] or can be processed into particles of tunable size and morphology [51,55,59,60,61,62], altering their loading capacity and release profile. For skin tissue engineering and wound-healing applications, utilizing PDA in particle form rather than as a coating provides greater flexibility on the timing of particle incorporation into the engineered skin fabrication process and allows the ability to add the drug-loaded particles immediately prior to grafting.

Release of bFGF from particles and scaffolds was shown to improve EC migration and proliferation in vitro [22,63,64,65] and accelerate angiogenesis in vivo [22,64,65,66,67,68]. The bFGF dose required to enhance vascularization and improve wound healing varies substantially based on model and delivery mode/carrier. In vitro models commonly utilize between 0.1 and 100 ng/mL [29,30,69]. In a bFGF-loaded fibrin matrix, tubule length increased in a dose-dependent manner, with a 2-fold increase in tubule length in the 100 ng/mL group at day 14 compared to 10 ng/mL [69]. When topically administered to enhance wound healing, effective doses ranging from ~0.1–10 µg/cm^2^ have been reported [32,33,34,36]. Recent publications [22,70,71,72] utilizing a delivery vehicle for sustained localized delivery of bFGF to subcutaneous and cutaneous wounds reported the use of total initial loading concentrations between 0.195 and 63.7 µg/cm^2^; however, daily in vivo bFGF release was not known. Delivery of bFGF from heparin-based sericin hydrogels at a total loading of ~0.35 µg/cm^2^ resulted in a 4-fold increase in vessel density in the wound compared to non-loaded gels [73]. The reported in vitro release profile showed a ~60% cumulative release by day 14; however, the in vitro release rate would likely be different than in vivo given the vastly different chemical and mechanical environment of the wound [73]. As the current study is a combined in vitro/in vivo study, initial loading concentrations between 0 and 100 µg/cm^2^ bFGF were utilized in an attempt to balance the anticipated “ideal” needs in both environments. We hypothesize that utilizing PDA microparticles to deliver bFGF at a dose between 10 and 100 µg/cm^2^ in engineered skin would localize the release of bFGF, protect bFGF from degradation in vitro and in vivo, and prolong the release of bFGF compared to a single dose. To test this hypothesis, human cell-based engineered skin was developed on electrospun collagen scaffolds that were seeded with PDA microparticles loaded with varying amounts of bFGF. Dermal contraction and ES morphology were first evaluated in vitro. ES was then grafted onto full-thickness wounds in immunodeficient mice and assessed for immune cell infiltration and vascularization.

## 2. Materials and Methods

### 2.1. PDA Microparticle Fabrication and bFGF Loading

Polydopamine (PDA) microparticles were formed via oxidative polymerization of dopamine monomers. Briefly, 120 mg of dopamine hydrochloride (Sigma-Aldrich, St. Louis, MO, USA) was added to 60 mL of water and stirred for 10 min. Sodium hydroxide (400 µL, 1 N) was then added to the solution and stirred for 90 min. The solution was then centrifuged at 13.3 k rpm for 15 min, the supernatant aspirated, and the remaining particles resuspended in water. Centrifugation and aspiration were repeated, and the remaining particles were lyophilized and stored at 4 °C.

Prior to bFGF loading, PDA microparticles were disinfected with sterile 70% ethanol at room temperature overnight. PDA microparticles were then centrifuged and rinsed with sterile water three times, as described above. Basic fibroblast growth factor (bFGF; ThermoFisher Scientific, Waltham, MA, USA) was reconstituted in a sterile solution of 0.1 *w*/*w*% bovine serum albumin (MP Biomedicals, Irvine, CA, USA) and heparin (Sigma-Aldrich) in water. Particles were split for the desired treatment groups, centrifuged, and loaded with bFGF overnight on a plate rocker at 4 °C. bFGF was loaded relative to PDA mass, where 100 ug of PDA was loaded with 0 (PDA+0bFGF), 10 (PDA+10bFGF), 50 (PDA+50bFGF) or 100 (PDA+100bFGF) ug bFGF. Particles were rinsed in sterile water three times and used immediately thereafter. For dermal template (DT) and engineered skin (ES) studies, particles were resuspended in Dulbecco’s Modified Eagle Medium (DMEM; ThermoFisher Scientific) with 1% antibiotic-antimycotic (Gemini BioProducts, West Sacramento, CA, USA).

### 2.2. Transmission Electron Microscopy (TEM)

Lyophilized PDA microparticles were diluted to a final concentration of 10 µg/mL in 18.2 MΩ MilliQ water, then sonicated using a micro-tip probe sonicator at 10% amplitude for 10 s with a 2 s ON/OFF cycle (Fisher Scientific Sonic Dismembrator 500, Thermo Fisher Scientific, Waltham, MA, USA). Solutions were applied dropwise (7 µL total) to a copper TEM grid, excess solution was blotted, and then the grids were imaged using a FEI Techni G2 Spirit (FEI Technologies Inc, Hillsboro, OR, USA).

### 2.3. Dynamic Light Scattering (DLS)

DLS analysis was performed using Zeta PALS, Zeta Potential & Particle Size Analyzer (Brookhaven Instruments, Nashua, NH, USA). All samples were diluted in deionized water to a concentration of 10 µg/mL. Samples were subjected to probe sonication, and 3 mL of each solution was added to a cuvette. Measurements were performed and reported as number-weighted intensity.

### 2.4. Zeta Potential

PDA microparticles were suspended at 10 µg/mL in deionized water (18.2 MΩ), and 2 mL of the solution was used to measure the zeta potential using Zeta PALS. Measurements were taken in triplicate and reported as average ± standard deviation.

### 2.5. bFGF Entrapment

The entrapment of bFGF by PDA microparticles was determined using an ELISA kit (R&D Systems, Minneapolis, MN, USA) and microplate reader (Synergy H1; Biotek Instruments, Santa Clara, CA, USA). Following the loading of bFGF, tubes of each treatment group (PDA+10bFGF, PDA+50bFGF and PDA+100bFGF) were centrifuged at 13.3 k rpm for 10 min, and the supernatant was collected. The pellets were washed two times, with the supernatant collected after each wash. The collected supernatant solutions were combined for each treatment group and analyzed by ELISA to estimate the amount of unbonded bFGF. The amount of loaded bFGF was quantified by subtracting the amount of unbonded bFGF from the total amount of bFGF divided by the total amount of bFGF, with the average % entrapped ± standard deviation reported for each group.

### 2.6. bFGF Release Kinetics

The bFGF release from PDA microparticles was performed by the direct suspension method. Briefly, 10 µg of each bFGF-loaded PDA microparticle group (PDA+10bFGF, PDA+50bFGF and PDA+100bFGF, n = 6) was added to 1.5 mL Eppendorf tubes and suspended in 1 mL of serum-free cell culture medium. The tubes were incubated in a shaking incubator (Incu-Shaker, Benchmark Scientific, Sayreville, NJ, USA) at 50 rpm and 37 °C. At incubation days 1, 3, 5, and 7, the tubes were centrifuged at 13.3 k rpm at 4 °C for 10 min and the supernatants were collected and stored at −80 °C. The pellets were then resuspended in fresh medium at each time point until day 7. The amount of bFGF released into the cell culture medium was quantified using an ELISA kit (R&D Systems), with average (ng/mL) ± standard deviation reported.

### 2.7. Fabrication of Dermal Templates

Electrospun collagen scaffolds were prepared as previously described [74]. Briefly, a 10% wt./vol. solution of bovine collagen type I (DSM Biomedical, Exton, PA, USA) and hexafluoroisopropanol (HFIP, Oakwood Chemical, Estill, SC, USA) was spun at a flow rate of 4 mL/hr (kDS 100; kD Scientific, Holliston, MA, USA), ~30 kV (Glassman High Voltage, High Bridge, NJ, USA), and a tip-to-ground distance of ~18 cm. Scaffolds were dehydrothermally crosslinked at 140 °C followed by chemical crosslinking with 5 mM N-(3-dimethylaminopropyl)-N′-ethylcarbodiimide hydrochloride (EDC; Sigma-Aldrich) in absolute ethanol for 24 h each. Scaffolds were then disinfected in sterile 70% ethanol and rinsed twice with PBS, five times with HEPES-buffered saline, and once in cell culture medium. PDA microparticles loaded with or without bFGF were applied onto the scaffolds at a density of 100 µg PDA microparticles/cm^2^ and incubated at 37 °C for one hour to facilitate attachment. As PDA is inherently adhesive to collagen largely through the catechol–OH and –NH_2_ functional groups as reported earlier [75], no additional chemical or physical treatment is needed to facilitate PDA microparticle attachment to the scaffolds. Scaffolds for control samples were incubated similarly without PDA microparticles. Four conditions were analyzed: control (no PDA), PDA+0bFGF, PDA+10bFGF and PDA+100bFGF. The PDA+50bFGF group was eliminated from further investigation as its release was similar to the PDA+100bFGF group.

Primary human dermal fibroblasts (HF) were cultured in DMEM and supplemented as previously described [74,76,77]. Cells were used at passage 2 for all experiments. DTs were constructed by seeding HF onto control and PDA microparticle-seeded scaffolds at 5E5 cells/cm^2^. Cell culture medium was changed daily.

### 2.8. Dermal Template Characterization

To quantify in vitro contraction, six 10 mm disks were collected from each dermal template (control, PDA+0bFGF, PDA+10bFGF and PDA+100bFGF). On days 1, 4, and 7, disks were removed from culture medium and photographed with a scale bar in the field of view. ImageJ v1.54c was used to quantify the area of each disk, and values are reported as average % area of initial ± standard deviation. On day 7, samples were embedded in OCT compound (ThermoFisher Scientific) for cryosectioning (7 µm thickness), for hematoxylin and eosin (H&E, ThermoFisher Scientific) staining and brightfield imaging (Leica DM2500 LED; Leica Microsystems, Boston, MA, USA). Representative H&E images and photographs at culture day 7 are reported.

Proliferation of fibroblasts within DTs as a function of bFGF concentration was quantified using an MTT assay (Sigma-Aldrich) as previously described [78]. Four 4 mm punches were collected from each DT (control, PDA+0bFGF, PDA+10bFGF, PDA+100bFGF) at days 1, 4, and 7. To evaluate and eliminate the interference of the PDA microparticles in the assay, 4 mm punches were also taken from control collagen scaffolds and PDA microparticle-seeded collagen scaffolds and included in the assay. These values were used to normalize the absorbance readings (590 nm) of control or PDA microparticle-containing DTs, respectively. The resulting values are reported as average absorbance ± standard deviation.

At culture day 7, biopsies were collected to quantify expression of genes encoding for α-SMA (ACTA2), COL1A1, and COL3A1 using qRT-PCR as previously described [79]. Gene expression was normalized to housekeeping gene GAPDH, and the 2 method was utilized to calculate the relative gene expression to the control group. Average normalized gene expression ± standard deviation are reported.

### 2.9. Fabrication of Engineered Skin with PDA Microparticles

Primary human keratinocytes (HK, p2) were cultured as previously described [74,76,77] in MCDB 153 medium. HK were seeded onto ~6.5 × 6.5 cm dermal templates at 1 × 10^6^ cells/cm^2^, four days after seeding HF. Engineered skin (ES) constructs were cultured in DMEM/F-12 (1:1, Sigma-Aldrich) supplemented with 1% antibiotic-antimycotic, 0.3% fetal bovine serum (Gemini BioProducts), 1% vol./vol. insulin-transferrin-selenium, 1 mM strontium chloride, (Sigma-Aldrich), 0.1 mM ascorbic acid-2-phosphate, 0.5 μg/mL hydrocortisone, 20 pM tri-iodothyronine, 1 ng/mL basic fibroblast growth factor, 5 ng/mL keratinocyte growth factor, and 10 μg/mL linoleic acid (ThermoFisher Scientific). One day after seeding with HK, ES were lifted to the air–liquid interface and medium was exchanged every other day. At culture days 5 and 10, four 4 mm punches were collected from each group (control, PDA+0bFGF, PDA+10bFGF, PDA+100bFGF) and an MTT assay was performed. Additional samples from each condition were collected at these timepoints, embedded in OCT and cryosectioned (7 µm). ES morphology was assessed via hematoxylin and eosin (H&E) staining with brightfield imaging. Additionally, immunohistochemical (IHC) staining was performed using rabbit monoclonal anti-Ki67 (#ab16667, Abcam, Cambridge, MA, USA) and mouse monoclonal anti-cytokeratin 15 (#ab80522, Abcam), followed by AlexaFluor^®^ secondary antibodies (ThermoFisher Scientific) and counterstaining with nuclear stain 4′,6-diamidino-2-phenylindole (DAPI; Sigma-Aldrich). Three fields of view (FOVs) for each of four samples per treatment group were imaged at 20× magnification using a Lecia Stellaris 5 confocal microscope. Representative brightfield and confocal images are shown. The number of Ki67-positive nuclei were counted in each FOV, and ImageJ was utilized to measure epidermal height (n = 3 measurements per FOV). FOV measurements were averaged for each sample, and average Ki67-positive epidermal nuclei/FOV and epidermal height ± standard deviation are reported.

### 2.10. In Vivo Assessment of ES Vascularization and Healing

All animal care and procedures followed National Institutes of Health guidelines and were approved by the Ohio State University Institutional Animal Care and Use Committee (#2017A00000021-R2). Full-thickness excisional wounds (2 × 2 cm) were created on the flanks of immunodeficient athymic mice (Preclinical Therapeutics Mouse Modeling Shared Resource, The Ohio State University James Comprehensive Cancer Center, Columbus, OH, USA). Eight to ten days after seeding HKs, PDA-containing ES grafts (PDA+0, 10 or 100 µg bFGF/cm^2^) were cut to 2 × 2 cm and sutured (Ethilon 6-0 P-3 1698G, Ethicon, Johnson & Johnson Medical Technologies, Irvine, CA, USA) to the wound site with overlying N-terface^®^ non-adherent dressing (Winfield Laboratories, Inc., Richardson, TX, USA). Gauze coated with Neosporin^®^ (Johnson & Johnson Consumer Companies, Inc., New Brunswick, NJ, USA) was secured to the grafts with a tie-over stent. Sites were covered with TegadermTM and CobanTM (3MTM, St. Paul, MN, USA). Mice and dressings were assessed daily for the duration of the study [80,81]. At post-grafting days 4, 7, and 14, six to nine animals per treatment group (Table S1) were undressed and photographed with a Nikon D7200 digital camera, with a scale bar and color balance card in the field of view. Animals were anesthetized with isoflurane while the graft sites equilibrated with the environment for 10 min prior to measuring for transepidermal water loss (TEWL) with a Tewameter^®^ TM300 probe (Courage + Khazaka Electronic GmbH, Köln, Germany). Average TEWL (g/hm^2^) ± standard deviation are reported. Animals were then euthanized for tissue collection (Table S1) and gross tissue adhesion/engraft along with vascularization scored. Tissue adhesion to the wound bed: 0 (easy to remove), 1 (easy to remove with partial incorporation at the edges), 2 (moderately easy to remove with full incorporation at the edges), 3 (moderately difficult to peel off, incorporation at the edges and partially along all wound bed), 4 (difficult to peel off, almost complete engraftment). Blood vessel presence within the graft/wound bed: 0 (little to no vessels observed, no bleeding during tissue removal), 1 (small number of vessels present, minimal punctate bleeding), 2 (numerous vessels present of varying size, modest bleeding during tissue removal), 3 (numerous large vessels observed, significant bleeding during tissue removal). Tissue samples were embedded in OCT, cryosectioned, stained with H&E, and imaged via brightfield microscopy. Additional samples were collected for PCR analysis as described above.

### 2.11. Immunohistochemistry for Vascularization and Inflammatory Cells

Vascularization was assessed via immunostaining at post-grafting days 7 and 14 with rat monoclonal anti-mouse CD31 (#557355, BD Biosciences, Franklin Lakes, NJ, USA), rabbit polyclonal anti-collagen IV (#ab6586, Abcam), rabbit polyclonal anti-von Willebrand Factor (VWF, #ab6994, Abcam), and mouse monoclonal anti-alpha-smooth muscle actin (αSMA, # 14-9760-82, ThemoFisher Scientific). To reduce non-specific staining of αSMA, a mouse-on-mouse immunodetection kit (#BMK-2202, Vector Laboratories Inc., Burlingame, CA, USA) was utilized according to the manufacturer’s protocol for blocking and secondary antibody incubation. The presence of neutrophils (rat monoclonal anti-neutrophil 7/4, #ab53457, Abcam) and macrophages (rabbit monoclonal anti-F4/80, #ab300421, Abcam) was assessed at post-grafting days 4 and 7. All antibodies were detected using appropriate AlexaFluor^®^ secondary antibodies and counterstained with DAPI. Sections were imaged at 20× magnification using a Lecia Stellaris 5 confocal microscope. Two discrete fields of view, through the depth of the dermis, were imaged for each sample. Presence of CD31, VWF, neutrophils, and macrophages was quantitatively analyzed using ImageJ to calculate the area positive for each antibody in the dermis of each sample, normalized to the total area of dermal and subdermal tissue. Values are reported as average % area positively stained per FOV ± standard deviation.

### 2.12. Statistical Analyses

Prism (GraphPad Software, Boston, MA, USA) was used to analyze all data. The differences among treatment groups were evaluated with a one-way analysis of variance (ANOVA) with Tukey’s post hoc test. Statistical significance was established with a *p* value < 0.05.

## 3. Results

### 3.1. Characterization of PDA Microparticles

**Figure 1:**
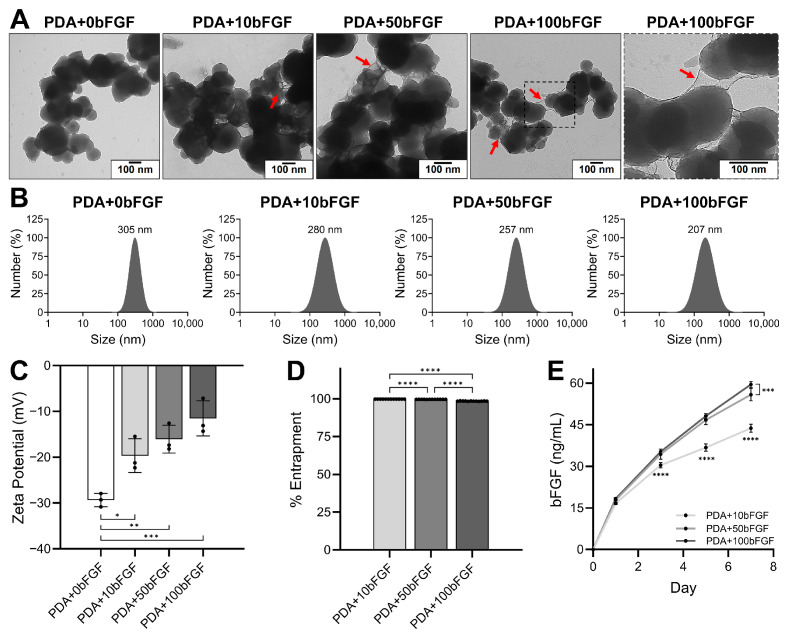
Characterization of PDA microparticles unloaded (PDA+0bFGF) and loaded with bFGF at 10 (PDA+10bFGF), 50 (PDA+50bFGF), and 100 (PDA+100bFGF) µg bFGF for every 100 µg PDA. (**A**) Representative TEM images, displaying regions of bFGF on the surfaces of loaded PDA microparticles (red arrows). (**B**) DLS analysis of PDA microparticles, with size values indicated at the peak intensities for each treatment group. (**C**) Zeta potential of PDA (n = 3). (**D**) Percent entrapment of bFGF by PDA (n = 12). (**E**) bFGF release from PDA microparticles over time (n = 6, **** indicates PDA+10bFGF vs. PDA+50bFGF and PDA+100bFGF). * *p* < 0.05, ** *p* < 0.01, *** *p* < 0.001, **** *p* < 0.0001.

When visualized with TEM, PDA particle size distribution and morphology were similar before and after treatment with BSA/heparin alone or with increasing amounts of bFGF (Figure 1A). An interconnecting network was visible around and between PDA microparticles loaded at a ratio of 100 µg bFGF:100 µg PDA (PDA+100bFGF), and this presence decreased with decreases in bFGF. DLS analysis revealed an inverse relationship between microparticle size and bFGF concentration (Figure 1B). bFGF loading resulted in significant increases in zeta potential of the PDA microparticles (for PDA+0bFGF, *p* < 0.05 vs. PDA+10bFGF, *p* < 0.01 vs. PDA+50bFGF, and *p* < 0.001 vs. PDA+100bFGF, Figure 1C). Entrapment of bFGF was significantly different among the three groups, slightly decreasing with increasing concentration of bFGF, though entrapment rates were high for all groups, with 99.81%, 99.64%, and 98.47% for PDA+10bFGF, PDA+50bFGF, and PDA+100bFGF, respectively (Figure 1D).

Release of bFGF from PDA microparticles was noticeably lower in the PDA+10bFGF group (Figure 1E) and remained significantly lower than the PDA+100bFGF group at all time points. At culture day 7, all groups were significantly different from each other, though only a small difference was observed between the PDA+50bFGF and PDA+100bFGF groups, with averages of 55.82 and 59.52 ng/mL, respectively.

### 3.2. Dermal Template Development and Contraction

**Figure 2:**
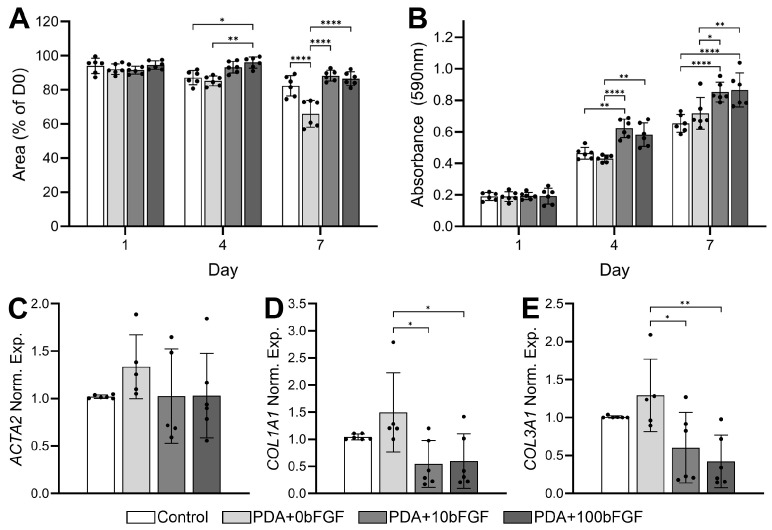
In vitro assessment of fibroblast-containing dermal templates seeded with bFGF-loaded PDA microparticles (PDA+10bFGF or PDA+100bFGF) compared to control and PDA microparticles alone (PDA+0bFGF). Quantification of dermal template contraction (**A**, n = 6) and metabolic activity (**B**, n = 6) at days 1, 4, and 7. Expression of genes ACTA2 (**C**), COL1A1 (**D**), and COL3A1 (**E**) in dermal templates at culture day 7 (n = 5–6). * *p* < 0.05, ** *p* < 0.01, **** *p* < 0.0001.

Fibroblast-seeded DTs (Figure S1A) were developed without PDA microparticles (control), with non-loaded PDA microparticles (PDA+0bFGF), or with PDA microparticles loaded with bFGF at 10 µg/cm^2^ (PDA+10bFGF) or 100 µg/cm^2^ (PDA+100bFGF). The PDA microparticles were mostly confined to the upper third of the scaffold; similarly, fibroblast densities were greatest at the surface of the scaffold and decreased in the deeper regions (Figure S1A). The PDA group without bFGF contracted the most by day 7 (Figure S1B and Figure 2A). This response appeared to be offset by the presence of bFGF as the area of each sample for the PDA+10bFGFor PDA+100bFGF groups was similar to or greater than that of the PDA+0bFGF group at day 7 (Figure 2A). Proliferation was also greatest in DTs containing bFGF-loaded PDA microparticles with significant increases in proliferation in the PDA+10bFGF and PDA+100bFGF groups vs. controls (*p* < 0.0001) (Figure 2B). Though PDA+0bFGF DTs contracted more significantly than the other groups, expression of ACTA2 in the DTs was not significantly altered by the presence of the PDA-MPs or bFGF (Figure 2C). In contrast, COL1A1 and COL3A1 expression was significantly increased in the PDA+0bFGF group compared to both bFGF-loaded groups (Figure 2D,E).

**Figure 3:**
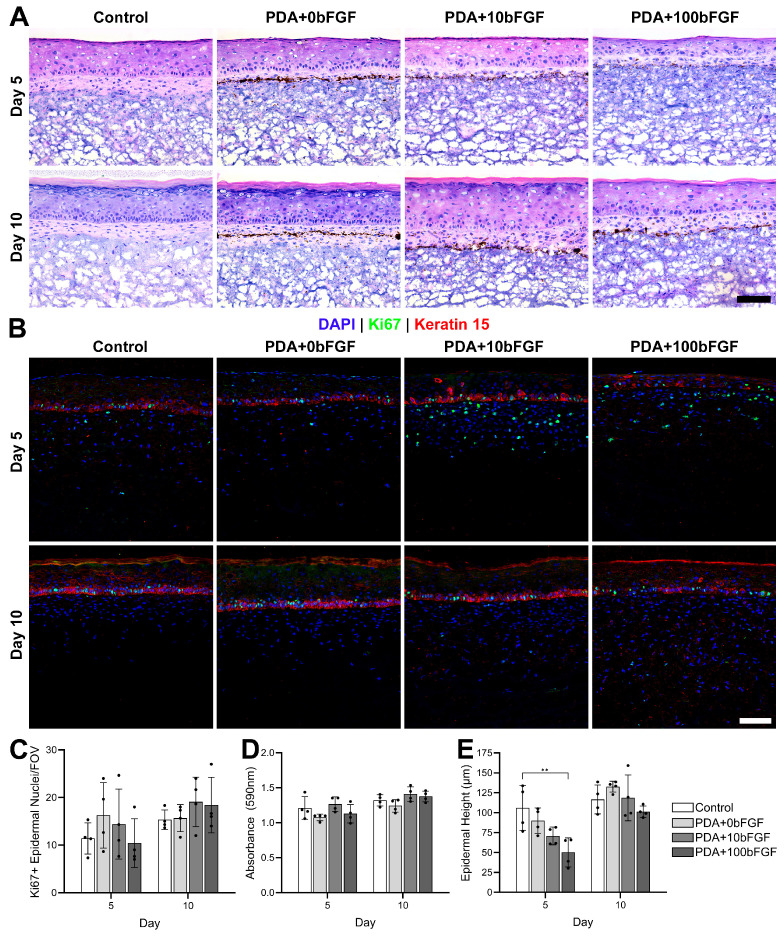
In vitro assessment of engineered skin seeded with bFGF-loaded PDA microparticles (PDA+10bFGF or PDA+100bFGF) compared to control and PDA microparticles alone (PDA+0bFGF) at culture days 5 and 10 post-keratinocyte seeding. Representative images (scale bars = 100 µm) for H&E (**A**) and immunohistochemical (IHC) staining (**B**) for Ki67 (green) and keratin 15 (red), with DAPI counterstain. Quantitative analysis (n = 4 samples, 3 FOVs/sample) of Ki67^+^ epidermal nuclei per field of view (**C**), metabolic activity (**D**), and height of the epidermis ((**E**), 3 measurements for each FOV). ** *p* < 0.01.

All of the groups of ES had a well-formed dermis and epidermis, with the PDA microparticles positioned within the dermis but below the dermal–epidermal junction (Figure 3A). Basal keratinocytes were present along the dermal–epidermal junction but appeared to be better organized in the control and PDA+0bFGF groups (Figure 3A). Numbers of Ki67^+^ keratinocytes were slightly higher on average in the bFGF-loaded PDA microparticle groups; however, this difference was not statistically significant (Figure 3B,C), and engineered skin fabricated with bFGF-loaded PDA microparticles showed similar levels of total skin metabolism among both controls and bFGF concentrations (Figure 3D). At culture day 5, the height of the epidermis trended downward with the addition of PDA and bFGF, with a significant difference determined between control and PDA+100bFGF groups (*p* < 0.01), though all groups were similar at culture day 10 (Figure 3E).

### 3.3. Engineered Skin Anatomy, Vascularization, and Engraftment

**Figure 4:**
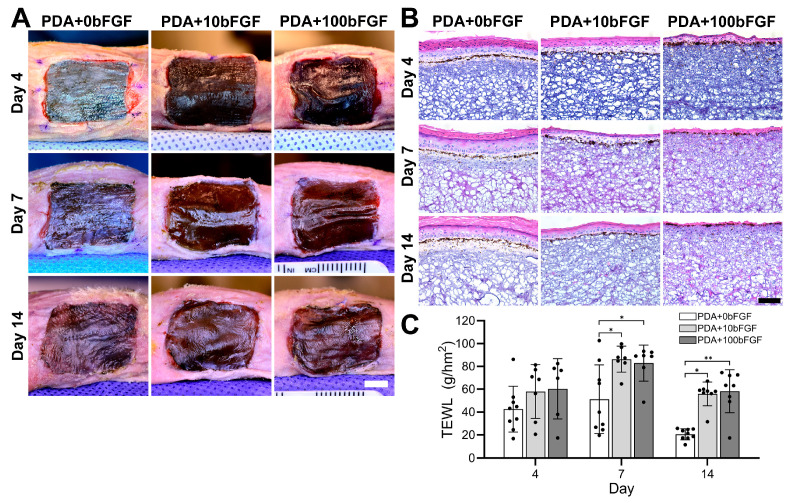
In vivo assessment of ES grafts fabricated with bFGF-loaded PDA microparticles (PDA+10bFGF or PDA+100bFGF) compared to PDA microparticles alone (PDA+0bFGF). Representative photographs (**A**, scale bar = 500 µm), representative H&E images (**B**, scale bar = 100 µm), and transepidermal water loss analysis (**C**, TEWL, PDA+0bFGF: n = 9; PDA+10bFGF: n = 7 for days 4 and 7, n = 8 for day 14; PDA+100bFGF: n = 6 for day 4, n = 7 for day 7, n = 8 for day 14) of grafts at days 4, 7 and 14 post-grafting to immunodeficient mice. * *p* < 0.05, ** *p* < 0.01.

In an effort to reduce the number of animals required, the PDA+50bFGF group assessed in PDA microparticle characterization was eliminated because little difference was observed in total bFGF release and bFGF release rate (Figure 1E) between the PDA+50bFGF and PDA+100bFGF groups. At 4 days post-grafting to mice, bFGF-containing grafts appeared better integrated with the wound at the borders (Table S2), although these were less keratinized than PDA control grafts without bFGF (Figure 4A,B). Note that the grafts appear pigmented due to the presence of PDA microparticles rather than melanin. Over time, grafts in the PDA control group further integrated with the host mouse skin, although some graft dehiscence was noted along the cranial and caudal edges of the bFGF-containing grafts (Table S2). Upon excision from the wound bed and examination, the tissue under the PDA+100bFGF grafts appeared vascularized macroscopically at day 4, with the majority of the visible vessels localized to the center of the graft site (Table S2). In contrast, the PDA+0bFGF and PDA+10bFGF groups had little to no granulation tissue under the graft with very few visible vessels. The trend was generally observed over the sample collection period that vascularized tissue presence increased with bFGF treatment concentration, with the least observed under PDA grafts and the most under PDA+100bFGF grafts, which displayed many blood vessels at day 14 (Table S2).

Histological assessment via H&E staining revealed expected development of PDA microparticle-containing ES grafts, with a discrete epidermal compartment and a prominent stratum corneum (Figure 4B). The PDA+10bFGF and PDA+100bFGF grafts displayed a less developed epidermis in the center of the graft co-localized to the area where large vessels were visible. Epidermal development was most affected in PDA+100bFGF grafts, with a very thin epidermis present at day 14 and significant amounts of subdermal inflammatory cells present. Transepidermal water loss was used to quantify the development of the epidermal barrier, with barrier function inversely related to transepidermal water loss values. Despite all groups having a similar epidermal barrier at day 4, water loss was significantly elevated in bFGF-containing grafts at days 7 and 14 post-grafting, which correlated with the visual observations of epidermal thinning (Figure 4C).

**Figure 5:**
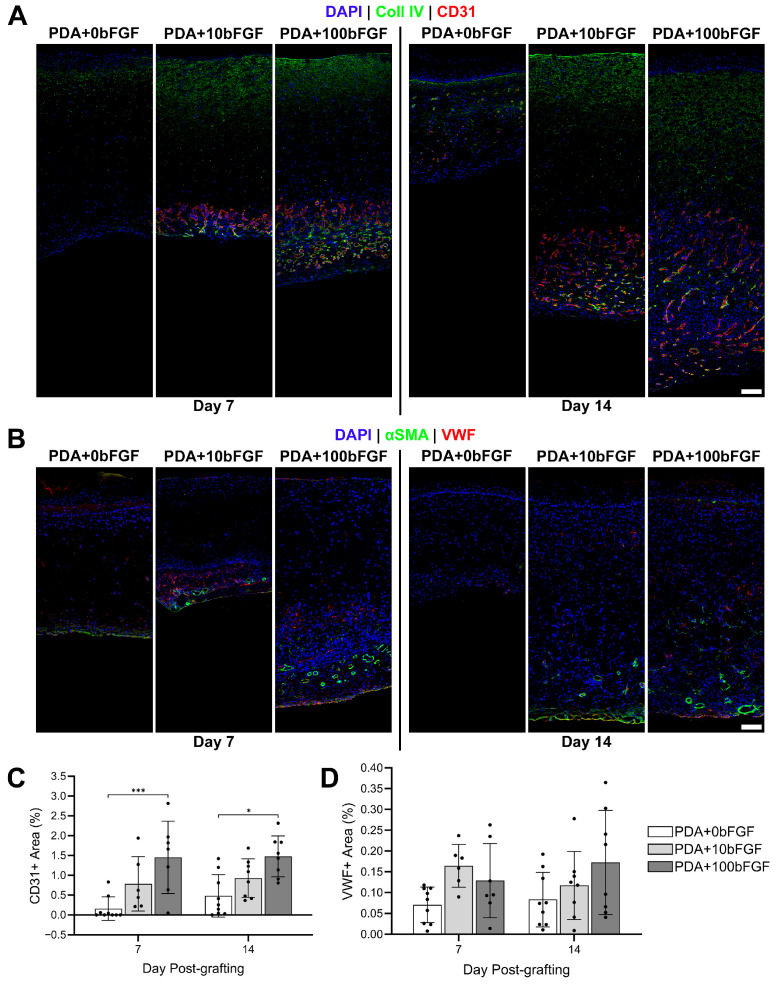
IHC localization of vascularization in ES grafts fabricated with bFGF-loaded PDA microparticles (PDA+10bFGF or PDA+100bFGF) compared to PDA microparticles alone (PDA+0bFGF). (**A**) IHC staining for collagen IV (green) and CD31 (red). (**B**) IHC staining for alpha-smooth muscle actin (αSMA, green) and von Willebrand Factor (VWF, red). DAPI was used as counterstain for nuclei (A&B, blue). Scale bars = 100 µm. Quantitative analysis of positive area of CD31 (**C**) and VWF (**D**) for each sample (PDA+0bFGF: n = 9; PDA+10bFGF: n = 6 for day 7, n = 8 for day 14; PDA+100bFGF: n = 7 for day 7, n = 8 for day 14; 2 full dermal depth images averaged per sample). * *p* < 0.05, *** *p* < 0.001.

Immunohistochemical staining revealed an increased number of blood vessels in bFGF-containing grafts at post-grafting day 7, which were visualized by positive CD31 (Figure 5A) and αSMA (Figure 5B) staining in intense, circular shapes primarily localized to the interface between the graft and the wound bed. Increased endothelial cell presence correlated with bFGF concentration and was significantly increased in PDA+100bFGF grafts over PDA+0bFGF grafts at days 7 and 14 post-grafting (Figure 5C). VWF was colocalized with some of these αSMA^+^ vessels, though it was also present outside of these structures, deep within the dermal region, suggesting localized or non-organized endothelial cells. Little positive staining for αSMA or VWF was observed in PDA+0bFGF samples, and vessels were noted in only a small number of regions of the grafts. However, when VWF was quantified and normalized to the total analyzed area, no significant differences between the groups were determined (Figure 5D). At day 14, little change from day 7 was noted in αSMA and VWF presence in PDA control grafts. In PDA+10bFGF and PDA+100bFGF grafts, αSMA was still localized to the subdermal region at day 14, though VWF was increased in the dermal compartment. VWF^+^ area in PDA+100bFGF grafts was still significantly higher than in PDA+0bFGF grafts at day 14 and, again, was more variable.

**Figure 6:**
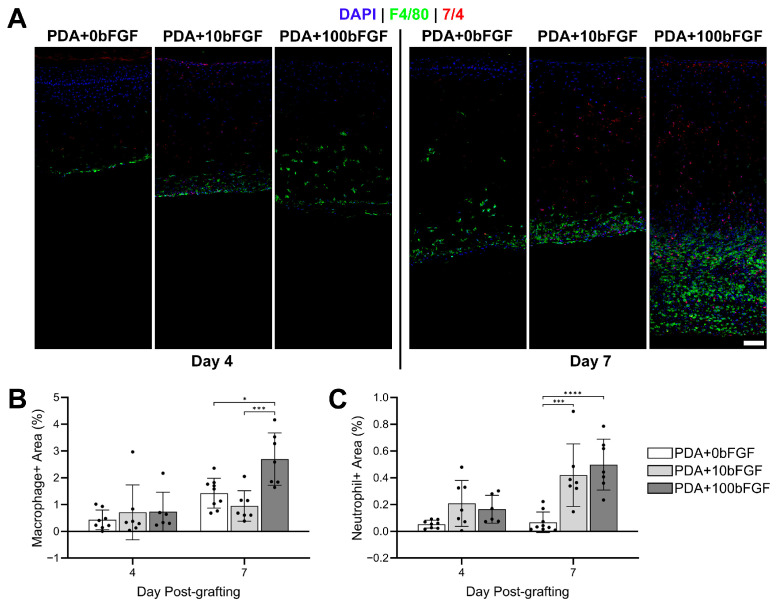
IHC localization of inflammatory cells in ES grafts fabricated with bFGF-loaded PDA microparticles (PDA+10bFGF or PDA+100bFGF) compared to PDA micropaticles alone (PDA+0bFGF). (**A**) IHC staining for macrophages (green) and neutrophils (red). DAPI was used as counterstain for nuclei (blue). Scale bar = 100 µm. Quantitative analysis of positive area of macrophages (**B**) and neutrophils (**C**) for each sample (PDA+0bFGF: n = 9; PDA+10bFGF: n = 7; PDA+100bFGF: n = 6 for day 4, n = 7 for day 7; 2 full dermal depth images averaged per sample). * *p* < 0.05, *** *p* < 0.001, **** *p* < 0.0001.

Immunostaining for inflammatory cells showed that macrophages were localized to the deep dermal and subdermal regions of grafts at day 4 post-grafting (Figure 6A) and were similar in the positive area for all treatment groups (Figure 6B,C). Neutrophils were increased in bFGF-containing grafts and were seen throughout the dermis in many of these grafts (Figure 6B,C). At day 7, macrophages infiltrated the dermal compartment of many PDA+0bFGF grafts while primarily remaining localized to the subdermal region in bFGF-containing grafts. Macrophage^+^ area increased in all grafts but significantly so in PDA+100bFGF grafts compared to day 4 and to PDA+0bFGF and PDA+10bFGF grafts. Neutrophils were sparse and largely absent from the dermal compartment of PDA+0bFGF grafts, though they were spread through the dermis of many PDA+10bFGF grafts and all PDA+100bFGF grafts, and the neutrophil+ area was significantly increased in the bFGF-containing grafts.

Tissue samples from grafts were processed for quantitative real-time PCR analysis of gene expression. However, significant differences in RNA yield and quality were observed between the control and PDA groups (Figure S2), which we attribute to interference from PDA, leading to unreliable gene expression and, thus, an inability to compare gene expression among groups.

## 4. Discussion

The ability to load PDA microparticles with bFGF and the potential for such a technology to speed vascularization in engineered skin was explored in this study. Although PDA microparticles have not previously been used as a delivery vehicle for bFGF, a PDA coating has been used to entrap bFGF to the surface of alginate microspheres, which exhibited a substantial burst release in vitro and promoted angiogenesis in vivo [82]. Additionally, PDA coatings on other materials have been used to deliver bFGF for up to 10–14 days with minimal burst release and no reported toxicity [83,84,85]. In the current study, bFGF was adsorbed to PDA microparticles in a solution of heparin and BSA, as heparin facilitates binding through its strong, non-covalent interactions with PDA [86,87] and its high binding affinity for bFGF [88,89,90,91], while BSA aids in stabilizing bFGF [92] and PDA particles [61,63]. The reduction in hydrodynamic diameter with increased loading of bFGF was due to the strong electrostatic interaction between PDA and bFGF which facilitates particle compaction. This phenomenon is consistent with other findings where the charge-based interaction results in a reduction in the nanoparticle size [93]. There was no significant difference in zeta potential between bFGF-loaded groups, but a statistically significant positive trend was observed with increasing bFGF loading concentration compared to control. This suggests increased surface functionalization of PDA microparticles with increased presence of bFGF, as PDA [94,95], heparin [96], and BSA [94,97] have a negative net charge while bFGF has a positive net charge at a neutral pH. BSA coating of PDA nanospheres has been previously performed [94], and a net positive change in zeta potential was observed, though it was not statistically significant compared to uncoated PDA nanospheres. Entrapment of bFGF was high (>98%), even at an equal mass ratio of bFGF to PDA microparticles, and release of bFGF scaled over time with loading concentration. However, only a small difference in release was observed between the PDA+50bFGF and PDA+100bFGF groups at day 7, which could be reflective of a high concentration of bFGF released into the supernatant of these two groups.

When assessed in vitro, the metabolic activity of fibroblasts in dermal templates was significantly increased by exposure to bFGF, suggesting increased proliferation, which has been seen previously [98,99]. The addition of PDA microparticles alone did not affect metabolic activity, but it did significantly increase DT contraction, and αSMA gene expression was increased ~33% compared with control samples. It has been widely reported that PDA films increase cell-body spreading and cellular adhesion to surfaces without decreasing viability [100,101,102,103]. Additionally, the expression in αSMA was shown to be increased in the lungs of mice with induced pulmonary fibrosis three days following intra-venous administration of mesoporous PDA microparticles vs. a saline solution [104]. The addition of bFGF appeared to mitigate the contractile effects of PDA in this study. Significant decreases in contraction in fibroblast-containing collagen gels have been seen when as little as 1 ng bFGF/mL was added to the cell culture medium [99,105], and administration of bFGF to a living skin equivalent decreased expression of αSMA in fibroblasts [106].

ES development in vitro was relatively similar among groups, and no toxicity was found for the three concentrations of bFGF that were tested. However, by day 4 post-grafting to mice, differences in epidermal stratification and barrier function, graft integration, and host response were apparent in the bFGF-containing groups. While the graft periphery generally maintained a stratified epidermis, the center displayed poorer epidermal differentiation with a thick dermal component, and this was most pronounced in PDA+100bFGF grafts. This tissue was found to be dense with blood vessels as determined by immunostaining for CD31, VWF, and αSMA. Previous animal studies have also demonstrated the angiogenic potential of bFGF delivered from wound dressings [71,107], microspheres [72], and coacervates [70] using CD31+ quantification. Further, αSMA^+^ staining has been used to demonstrate an increase in vascular density at day 7 and day 17 following administration of microspheres [22] and coacervates [70], respectively. Interestingly, it was found that bFGF loading as low as 1 µg/cm^2^ for scaffolds [71] and 25.5 µg/cm^2^ for microspheres [72] promoted vascularization at day 14, though increasing the loading of microspheres to 63.7 µg/cm^2^ resulted in a significant increase in vascularization at day 10 [72]. A scaled response was also noted here, where implanted scaffolds containing PDA+100bFGF were more vascularized than those with PDA+10bFGF; however, an inflammatory response was also observed to increase with increased delivery of bFGF. We do not believe the inflammatory response was due to transplantation of human cells onto the athymic mouse host. The absence of T cells in this immunodeficient mouse model precludes an adaptive immune response but still permits an innate immune response [108]. The exuberant inflammatory response observed here is likely attributed to bFGF as controls without bFGF in the current study, and similarly constructed dermal scaffolds in previous studies [74] did not exhibit such robust inflammation. Epidermal barrier function is closely related to skin inflammation: for example, in atopic dermatitis, mutations affecting barrier proteins can increase skin inflammation, but relieving this excess skin inflammation can, in turn, help restore the epidermal barrier [109]. Inflammatory cytokines can directly influence gene expression in keratinocytes to disrupt epidermal stratification and disrupt barrier function [110]. Thus, the inflammatory response to high levels of bFGF observed in ES may impair epidermal barrier by interfering with keratinocyte differentiation. This is consistent with the morphological changes observed in vivo in ES with the highest bFGF dose. However, because bFGF has been shown to directly impact epidermal differentiation [111], it is possible that the combined effects of bFGF and inflammation contributed to disrupted epidermal barrier function in PDA+100bFGF ES grafts.

CD31 expression is commonly correlated with the presence of endothelial cells and, thus, vascularization. However, CD31 expression on immune cells has also been reported, possibly explaining CD31+ staining not associated with vascular structures within granulation tissue for bFGF-containing grafts [112]. Alternatively, isolated staining for CD31 outside of vessels may suggest the presence of endothelial cells outside of well-organized vessels and might indicate ongoing vasculogenesis. Correlating with the increased granulation tissue formation seen in bFGF-containing grafts, the presence of inflammatory cells, specifically macrophages and neutrophils, was significantly increased in bFGF-containing grafts at day 7 post-grafting, suggesting a substantial inflammatory response to bFGF. bFGF is known to enhance recruitment of monocytes and neutrophils [113], upregulate expression of endothelial cellular adhesion molecules for leukocytes [114], and influence macrophage polarization [115,116]. Long-term release of bFGF from PDA microparticles past 7 days was not assessed in vitro but was likely affected in vivo by the wound environment. Reactive oxygen species from infiltrating immune cells and other reactive compounds involved in the wound repair process could have accelerated the degradation of PDA microparticles and thus the release of bFGF [117]. This effect may have been amplified at the center of the grafts if the bFGF was localized, whereas, in vitro, bFGF was released into a large reservoir of cell culture medium and, in vivo, bFGF at the periphery may have been able to diffuse out of the graft. Possible support for this is the VWF observed outside of blood vessels in the lower dermis and subdermal tissue of the bFGF-containing grafts, as VWF has been shown to bind to bFGF via the heparin-binding domain [118], and this localization has been associated with an inflammatory state, where VWF binds and modulates the behavior of macrophages [119]. Inflammatory cells were observed in all grafts but were relatively increased in bFGF-containing grafts, and significantly so at day 7 post-grafting.

The inflammatory response may have also been affected by the state of the grafted cells as, at the time of grafting, fibroblasts and keratinocytes in the ES had been primed with bFGF for 12–14 and 8–10 days, respectively. In previous studies, rats were sub-dermally injected with solutions containing 5–200ng bFGF with 5 ug heparin or a control of heparin in culture media two days before the injection of various inflammatory stimuli, such as tumor necrosis factor (TNF), interferon (IFN), and macrophage inflammatory protein (MIP) [114]. Treatment of bFGF alone did not induce recruitment of monocytes, T cells, or PMNs, but pre-treatment with bFGF significantly increased the recruitment of monocytes, 155–212% of control, and PMN, 130–232% of control, to the subdermal injection sites. Additionally, bFGF alone was found to increase the expression of ICAM-1 and P-selectin vs. control. Further, co-injection of bFGF with inflammatory stimuli TNF and IFN significantly increased the expression of ICAM-1, P-selectin, and E-selectin vs. injection of just the inflammatory stimuli [114].

Analysis of ACTA2, COL1A1, and COL3A1 gene expression in vitro indicated differences among DT groups; however, there was substantial variability in gene expression levels in groups containing PDA microparticles compared with control DTs without PDA microparticles. This may reflect actual variability among samples, but this also may be confounded by PDA interfering with RNA isolation and purification. Analysis of gene expression in vivo was initially planned; however, there was a significant decrease in RNA yield in the PDA groups compared to the controls from excised tissues which precluded this analysis. Similar experiments have previously shown interference between nucleic acids and gold nanoparticles [120]. Additionally, many other reports have shown interactions between nucleic acids (RNA, DNA, miRNA) and polydopamine [95,121,122]. These studies have attributed the presence of catechol and amine groups in PDA to the high π-π stacking and electrostatic interactions with nucleic acids, especially at a low pH (like TriZol used for isolation). Therefore, it is possible that the poor yield of the isolated RNA is due to the nucleic acid binding to the PDA particles, which may not have been degraded during the two weeks post-treatment. Issues with poor yield were not noted in the isolation of RNA from the dermal templates; these matrices had a far lower cell density, and we believe that there was less interaction with the PDA during the isolation process.

A limitation of the current study is that the PDA+50bFGF group was not transplanted to mice for in vivo investigation. The decision to transplant only the PDA control (PDA+0bFGF), low (PDA+10bFGF), and high (PDA+100bFGF) groups was made to reduce the number of animals required. However, the selection of only two doses precludes the ability to fully define the dose–response relationship. Therefore, while we can conclude that the observed responses scaled with bFGF dose, we cannot determine whether these responses scale linearly with bFGF concentration.

## 5. Conclusions

Polydopamine microparticles were efficiently loaded with multiple concentrations of bFGF and utilized in the development of DTs and ES in vitro. The presence of bFGF offset the contraction observed in DTs with PDA microparticles alone, while increasing metabolic activity. Epidermal development was slightly delayed in ES with the highest concentration of bFGF, though epidermal stratification was achieved by the time of grafting onto immunodeficient mice. Unfortunately, a significant inflammatory response was noted in PDA+bFGF grafts, and it was primarily in these subdermal inflamed regions that increased vascularization was observed. This correlated with a decline in epidermal differentiation, which was not noted in grafts with PDA microparticles alone. These results suggest that PDA microparticles are a viable drug delivery vehicle for dermal templates and engineered skin, though lower concentrations of bFGF or perhaps a different growth factor should be investigated in future studies.

## Data Availability

The data presented in this study are openly available in Dryad at DOI: 10.5061/dryad.fn2z34v8q.

## Supplementary Materials

The following supporting information can be downloaded at: https://www.mdpi.com/article/10.3390/bioengineering13010110/s1, Table S1: Number of subjects euthanized at each time point for each treatment group; Figure S1: In vitro assessment of fibroblast-containing dermal templates seeded with bFGF-loaded PDA (PDA+10bFGF or PDA+100bFGF) compared to control and PDA alone (PDA+0bFGF); Table S2: Scoring of macroscopic tissue features during skin collection; Figure S2: PDA appears to affect quality and yield of RNA isolated from tissue samples.

## Abbreviations

The following abbreviations are used in this manuscript:

PDA	Polydopamine
bFGF	Basic fibroblast growth factor
DT	Dermal template
ES	Engineered skin
VEGF	Vascular endothelial growth factor
PDGF	Platelet derived growth factor
EC	Endothelial cells
DMEM	Dulbecco’s Modified Eagle Medium
TEM	Transmission Electron Microscopy
DLS	Dynamic Light Scattering
HFIP	Hexafluoroisopropanol
EDC	N-(3-dimethylaminopropyl)-N’-ethylcarbodiimide hydrochloride
HF	Primary human dermal fibroblasts
HK	Primary human keratinocytes
H&E	Hematoxylin and eosin
IHC	Immunohistochemistry
FOV	Field of view
VWF	von Willebrand Factor
αSMA	Alpha-smooth muscle actin
TNF	Tumor necrosis factor
IFN	Interferon
MIP	Macrophage inflammatory protein
PMN	Polymorphonuclear leukocyte
