# Supplementary material for: bFGF-Loaded PDA Microparticles Enhance Vascularization of Engineered Skin with a Concomitant Increase in Leukocyte Recruitment
Source: Bioengineering (Basel). 2026 Jan 16;13(1):110. doi: 10.3390/bioengineering13010110 (PMC12837753; doi:10.3390/bioengineering13010110)
Supplement: Supplementary file 1 [file bioengineering-13-00110-s001.zip › bioengineering-4081538-supplementary.pdf]

Day	PDA+0bFGF	PDA+10bFGF	PDA+100bFGF
4	9	7	6
7	9	7	7
14	9	8	8

Table S1. Number of subjects euthanized at each time point for each treatment group.

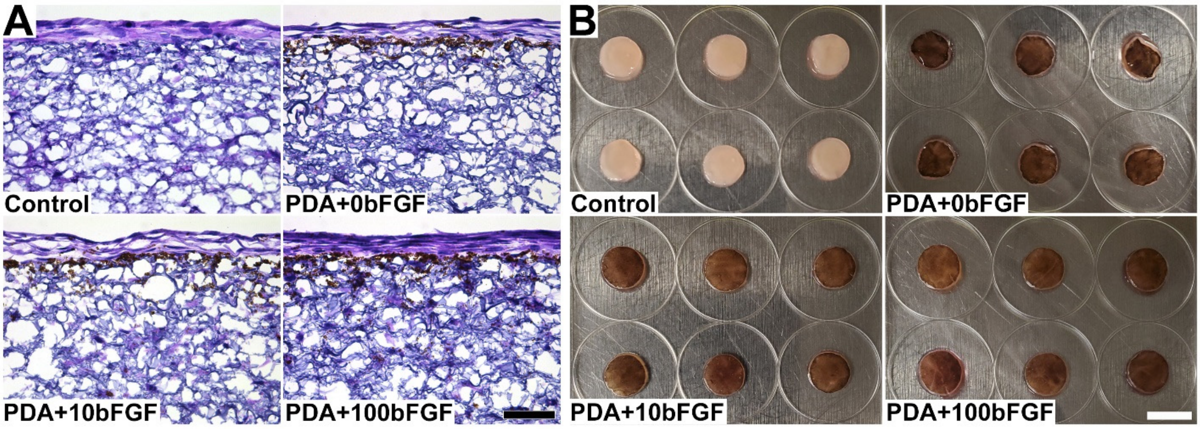

Figure S1. *In vitro* assessment of fibroblast-containing dermal templates seeded with bFGF-loaded PDA (PDA+10bFGF or PDA+100bFGF) compared to Control and PDA alone (PDA+0bFGF). A) Representative H&E images at culture day 7 (scale bar = 50 μm). B) Images of dermal template contraction at culture day 7 (scale bar = 1 cm).

Day	Adhesion/Engraftment			Vascularization		
	PDA+0bFGF	PDA+10bFGF	PDA+100bFGF	PDA+0bFGF	PDA+10bFGF	PDA+100bFGF
4	0.4 ± 0.7	0.9 ± 0.9	2.3 ± 1.2 [a,b]	0.0 ± 0.0	0.0 ± 0.0	1.8 ± 0.4 [a,d]
7	1.0 ± 1.2	2.6 ± 0.8	3.9 ± 0.4 [c]	0.2 ± 0.4	0.9 ± 0.9	2.1 ± 0.4 [c]
14	1.0 ± 1.2	3.1 ± 0.6 [a]	3.1 ± 0.6 [a]	0.3 ± 0.4	2.0 ± 1.2 [e]	2.5 ± 0.5 [a]

Table S2. Scoring of macroscopic tissue features during skin collection. Tissue adhesion to the wound bed: 0 (easy to remove), 1 (easy to remove with partial incorporation at the edges), 2 (moderately easy to remove with full incorporation at the edges), 3 (moderately difficult to peel off, incorporation at the edges and partially along all wound bed), 4 (difficult to peel off, almost complete engraftment). Blood vessel presence within the graft/wound bed: 0 (little to no vessels observed, no bleeding during tissue removal), 1 (small number of vessels present, minimal punctate bleeding), 2 (numerous vessels present of varying size, modest bleeding during tissue removal), 3 (numerous large vessels observed, significant bleeding during tissue removal). [a]$p < 0.01$ versus PDA+0bFGF at same time point, [b]$p < 0.05$ vs PDA+10bFGF at same time point, [c]$p < 0.001$ versus PDA+0bFGF at same time point, [d]$p < 0.01$ versus PDA+10bFGF at same time point, [e]$p < 0.05$ versus PDA+0bFGF at same time point.

Sample	Weight (mg)	Concentration (ng/µl)	A260 (Abs)	A280 (Abs)	260/280	260/230	RNA yield (ng/µl/mg)
Control 1	60.0	47.7	1.191	0.571	2.09	2.30	0.795
Control 2	53.7	29.4	0.736	0.375	1.96	2.32	0.547
Control 3	33.2	21.8	0.546	0.262	2.08	1.89	0.657
PDA 1	74.0	11.4	0.286	0.149	1.91	2.07	0.154
PDA 2	54.8	19.9	0.498	0.247	2.02	2.26	0.363
PDA 3	41.8	6.9	0.172	0.086	1.99	0.15	0.165

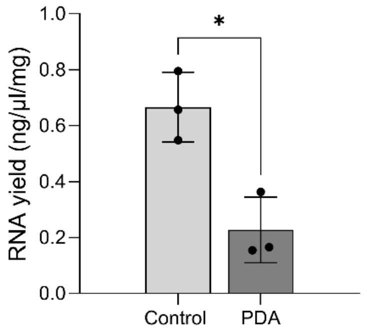

Figure S2. PDA appears to affect quality and yield of RNA isolated from tissue samples. Due to difficulties in purifying RNA for the current study, we analyzed engineered skin (ES) samples that were prepared identically (same cell strains and densities) and transplanted to mice for a related study but without PDA. RNA was isolated from 3 ES samples (*in vivo* day 14) without PDA grafted to mice (Controls 1-3) and samples from the current study containing PDA+10bFGF (PDA samples 1-3). The samples containing PDA initially had low 260/280 and 260/230 ratios (determined using a Nanodrop spectrophotometer; Thermo Fisher Scientific), indicating contamination. Note that 260/280 ratios of ~2.0 are generally considered sufficiently pure; lower ratios may indicate presence of protein or phenol contaminants that absorb at 280 nm (Mathieson and Thomas, 2013). The 260/230 rations are often higher than 260/280 ratios, but low levels may indicate contamination with carbohydrates, which absorb at 230 nm (Mathieson and Thomas, 2013). RNA from the PDA samples was extracted with phenol/chloroform and ethanol precipitated to clean up the samples, which improved the 260/280 ratios (see Table, above) but resulted in reduced yield. (Above) RNA concentrations and absorbance values reflecting the RNA purity and yield in Control and PDA-containing grafts at day 14 post-grafting. (Below) Concentration of RNA isolated per mg/sample (n = 3, *$p < 0.05$ via t test).

Reference:

Mathiesan W, Thomas GA. Simultaneously extracting DNA, RNA, and protein using kits: Is sample quantity and quality prejudiced? Anal Biochem 2013:433(1);10-18. PMID: 23068038.
